# Targeting ADAR1 in Cancer: Biology, Therapeutic Strategies, Challenges, and Limitations

**DOI:** 10.3390/ph19081250

**Published:** 2026-08-08

**Authors:** Carolyn N. Ashley, Emmanuel Broni, ChaNyah M. Wood, Simon Kaja, Sean W. Fanning, Scarlett Schuth, Whelton A. Miller

**Affiliations:** 1 Department of Medicine, Stritch School of Medicine, Loyola University Chicago, Maywood, IL 60153, USA; cashley1@luc.edu (C.N.A.); ebroni@luc.edu (E.B.);; 2 Department of Molecular Pharmacology & Neuroscience, Stritch School of Medicine, Loyola University Chicago, Maywood, IL 60153, USAskaja@luc.edu (S.K.); 3 Department of Ophthalmology, Stritch School of Medicine, Loyola University Chicago, Maywood, IL 60153, USA; 4 Department of Cancer Biology, Stritch School of Medicine, Loyola University Chicago, Maywood, IL 60153, USA

**Keywords:** small molecule inhibitors, ADAR1, RNA editing modulators, cancer therapeutics

## Abstract

Adenosine deaminase acting on RNA 1 (ADAR1) is a critical regulator of innate immune signaling and a pan-cancer therapeutic target. Through catalyzing adenosine-to-inosine (A-to-I) editing and editing-independent mechanisms, ADAR1 suppresses activation of dsRNA sensing pathways, including protein kinase R (PKR), melanoma differentiation-associated protein 5 (MDA5), and oligodenylate-synthetase (OAS) signaling, that are critical for maintaining cellular tolerance to endogenous RNAs. In a subset of tumors characterized by elevated interferon-stimulated gene (ISG) expression and dsRNA stress, this function creates a dependency on ADAR1 for survival, establishing a therapeutic vulnerability that can be exploited to induce viral mimicry in cancer cells and enhance anti-tumor immune responses. Here, we review the emerging landscape of ADAR1 modulators, organizing reported compounds into mechanistic classes including nucleoside analogs, catalytic inhibitors, Zα domain modulators, RNA substrate engagement inhibitors, indirect pathway regulators, and PROTACs. We evaluate molecules within these classes with a focus on their mechanisms of action and experimental validation. We further discuss the challenges associated with distinguishing direct inhibition of ADAR1 activity from broader effects on RNA metabolism and innate immune activation. Finally, we highlight the therapeutic potential of ADAR1 targeting defined cancer subsets and examine combination strategies that leverage ADAR1 inhibition for improved sensitivity to current cancer therapeutics. Overall, this review outlines key considerations for the development of selective therapies targeting ADAR1.

## 1. Introduction

Members of the ADAR enzyme family, ADAR1 and ADAR2, catalyze adenosine-to-inosine (A-to-I) editing within double-stranded RNA (dsRNA), collectively modifying hundreds of millions of RNA sites across the transcriptome [1]. This editing contributes to diverse biological processes, including regulation of gene expression and diversification of oncogenic and tumor-suppressive proteins through mechanisms like direct amino acid recoding, RNA interference, and alternate splicing modulation [2]. RNA editing independent mechanisms influenced by protein–protein and protein–RNA interactions also exist that contribute to ADAR functions [3]. Although ADAR family members share a high sequence similarity, particularly in the shared double-stranded RNA binding domains (dsRBDs) and a catalytic deaminase domain (CDD), they exhibit distinct functional roles, substrate specificities, and protein–protein interaction profiles driven by isoform-specific domains, sequence variation, and subcellular localization [4].

ADAR1 is uniquely positioned at the interface of RNA biology and innate immunity, where it demonstrates considerable functional versatility through its two isoforms, ADAR1p150 and ADAR1p110 [5]. Both ADAR1 overexpression and increased ADAR1 RNA editing have been observed in numerous cancers, and consistent with the diverse functional roles of ADAR1 influence a range of cancer-promoting pathways [6,7,8,9,10,11,12,13,14,15]. ADAR1p110, controlled by a constitutive promoter, regulates the resolution of telomeric repeat R-loops contributing to sustained proliferation capacity and genome stability within telomerase-reactivated cancers [16]. ADAR1p150, controlled by an interferon inducible promoter, is a critical node upstream of MDA5, PKR, ZBP1, and OAS-RNase L pathways, where its editing, largely of repetitive genomic elements like inverted Alu sequences, and editing-independent activities limit activation of cytosolic RNA sensors, highlighting ADAR1 as a critical suppressor of innate immune activation [17,18]. This regulatory role of ADAR1 in innate immunity gives rise to a context-dependent vulnerability to ADAR1 loss. The concept of ADAR1 dependency is where a subset of tumors relies on ADAR1 to suppress endogenous dsRNA sensing to maintain cellular viability [19]. In these contexts, when ADAR1 is depleted, a viral mimicry response is induced resulting in increased interferon signaling, translational inhibition, and loss of cell viability, positioning ADAR1 as a central regulator of tumor cell fitness [20,21].

ADAR1 is a compelling and complex therapeutic target due to its critical role in regulating endogenous nucleic acid sensing and maintaining cancer cell survival. Induction of viral mimicry through ADAR1 inhibition offers a promising strategy for selectively targeting cancer cells primed for ADAR1 sensitivity and to enhance immunotherapy responses. This therapeutic potential has driven increasing interest in development of ADAR1-directed and immunomodulatory strategies. Although most ADAR1-targeting approaches remain in preclinical development, selected strategies have begun advancing toward clinical evaluation [22,23]. Furthermore, emerging evidence from studies of immunomodulatory therapies has highlighted ADAR1 loss as a mechanism that can enhance anti-tumor responses, suggesting opportunities for therapeutic combinations that exploit ADAR1 dependency [23,24,25,26,27]. However, challenges for developing ADAR1-targeted strategies include achieving selective inhibition, distinguishing on-target effects from broader nucleotide metabolism perturbations, and understanding context-specific responses across cancer types. In this review, we examine the current explorations of strategies targeting ADAR1, with a focus on different mechanistic classes of modulators, their modes of action, and the experimental frameworks used to evaluate their activity. We further discuss how ADAR1 targeting can be applied to specific cancer vulnerabilities and highlight key challenges that must be addressed to translate ADAR1 biology into clinically effective interventions.

## 2. ADAR1 as a Context-Dependent Regulator of Cancer Cell Survival

**Figure 1:**
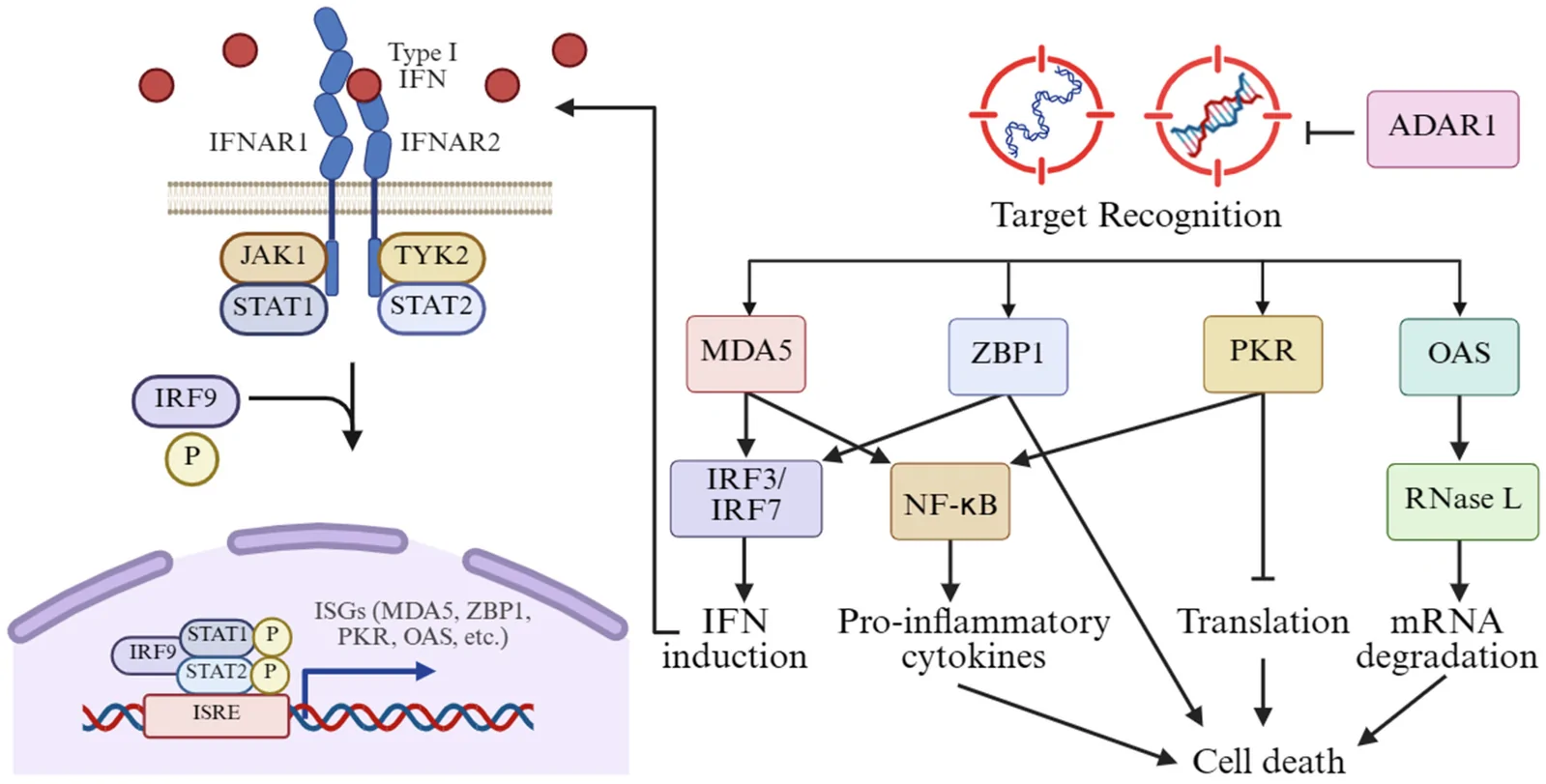
Mechanisms underlying ADAR1-mediated suppression of innate immune activation and cancer cell survival. ADAR1 prevents recognition of endogenous dsRNA and Z-DNA/Z-RNA structures by innate immune sensors including melanoma differentiation-associated protein 5 (MDA5), Z-DNA binding protein 1 (ZBP1), protein kinase R (PKR), and oligodenylate-synthetase (OAS). Loss or inhibition of ADAR1 results in activation of these pathways, promoting downstream signaling events that contribute to cancer cell death. ADAR1 inhibition also induces type I interferon (IFN) signaling, leading to increased expression of interferon-stimulated genes (ISGs), including innate immune sensors that further amplify the response.

**Figure 2:**
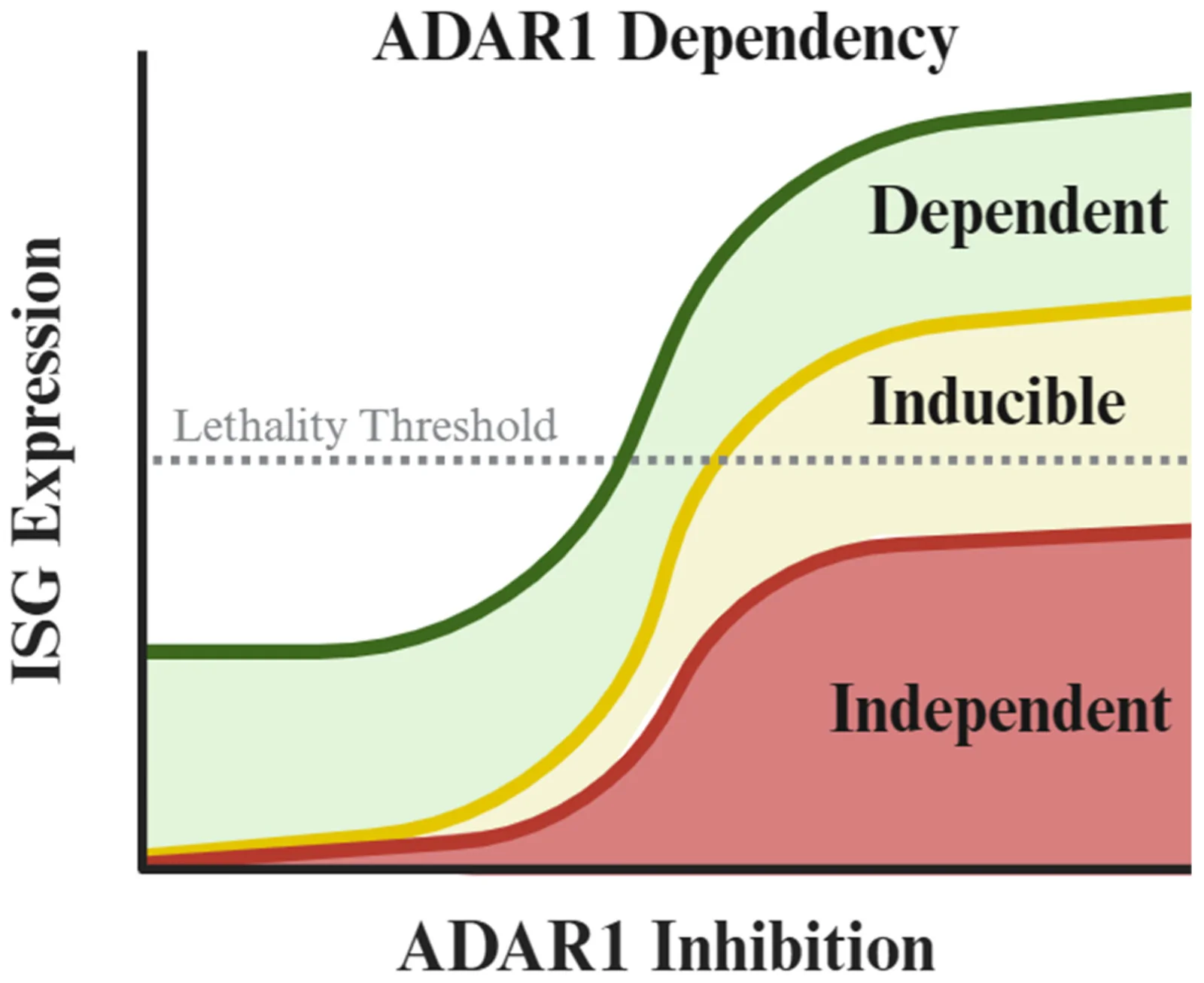
The landscape of ADAR1 dependency. Created in BioRender. Miller, W. (2026) https://BioRender.com/8va18rp.

ADAR1 dependency defines a subset of tumors that exist in a chronically interferon-stimulated state with an accumulation of immunogenic dsRNAs, rendering them reliant on ADAR1 to suppress excessive innate immune activation (Figure 1) [19]. In these contexts, loss of ADAR1 triggers a strong viral mimicry response pushing these cells over a lethal threshold characterized by more interferon signaling, translational inhibition, and cell death (Figure 2) [19,20,21]. In ADAR1-dependent TNBC models, loss of ADAR1 leads to pronounced phenotypes of cell cycle arrest and apoptosis, highlighting a strong requirement for ADAR1 in sustaining proliferation and survival [20]. This phenotype has been observed in multiple tumor types including cell lines of prostate cancer, non-small-cell lung cancer (NSCLC), head and neck cancer, ovarian cancer, and pancreatic cancer [19,20,21].

ADAR1-independent cells have a low basal ISG signature, where cells remain viable after ADAR1 loss (Figure 2) [21]. In A549 and H1299, two NSCLC cell lines reported to be ADAR1-independent; ADAR1 knockdown could still affect anti-tumor activities resulting in decreased cell growth, proliferation, and tumor growth, but not to the degree expected in ADAR1-dependent cell lines [10,21]. These findings indicate that ADAR1 contributes broadly to tumor biology but highlights a subset of cancers reliant on ADAR1 for survival. Notably, lethality can be induced in these ADAR1-independent cells by priming the cells using IFNs or epigenetic therapeutics to increase IFN signaling or increase accumulation of immunogenic dsRNAs (Figure 2) [21,28].

**Figure 3:**
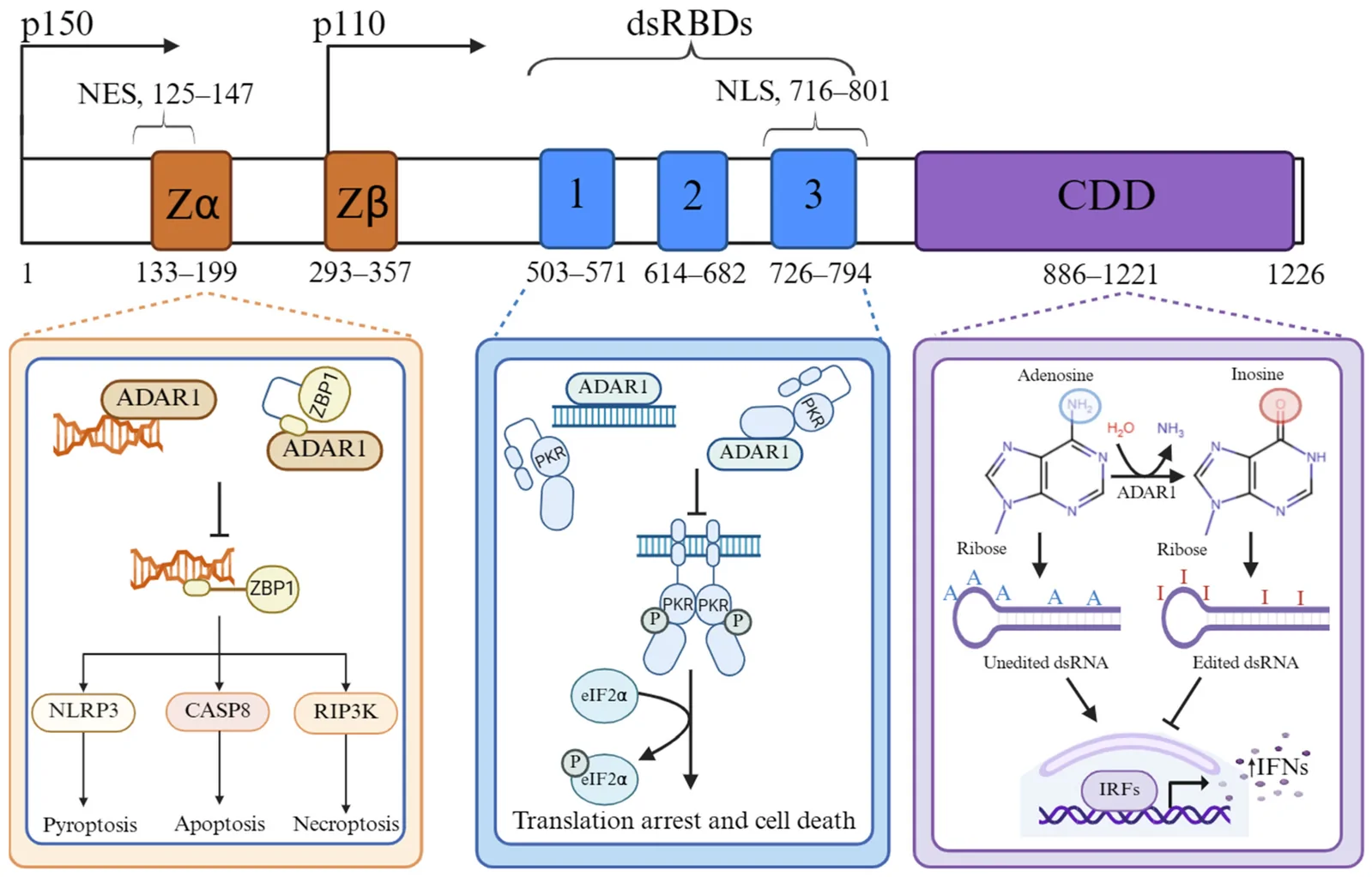
ADAR1p150 and ADAR1p110 domain architectures and functional mechanisms of ADAR1. The Zα domain of ADAR1p150 binds Z-RNA/DNA structures and can interact with ZBP1, preventing ZBP1-mediated sensing and downstream PANoptotic cell death. DsRBDs competitively bind dsRNA and engage in protein–protein interactions that limit activation of dsRNA sensors such as PKR, thereby inhibiting eIF2α phosphorylation and translational arrest. The CDD mediates adenosine-to-inosine editing of dsRNA; in the absence of editing, unmodified dsRNA activates innate immune signaling pathways, promoting IRF nuclear translocation and IFN production. NES, nuclear export signal; NLS, nuclear localization signal; dsRBD, double-stranded RNA binding domain; CDD, catalytic deaminase domain; ZBP1, Z-DNA binding protein 1; NLRP3, NOD-, LRR-, and pyrin domain-containing protein 3; CASP8, caspase 8; RIP3K, receptor-interacting serine/threonine-protein kinase 3; PKR, protein kinase R; eIF2α, eukaryotic translation initiation factor 2 subunit alpha; IRF, interferon regulatory factors; IFN, interferons; A, Adenosine; I, Inosine. Created in BioRender. Miller, W. (2026) https://BioRender.com/mte5du6.

ADAR1 dependency is not uniform and exists along a spectrum. Substantial heterogeneity exists across and within tumor lineages, suggesting that tumor sensitivity to ADAR1 loss is shaped by multiple factors at least in part by differing levels of ISG expressions (e.g., MDA5 and PKR) and immunogenic dsRNAs [19,29]. Other differences that contribute to cellular variability include varying expression levels of inflammatory pathway components between cell lines and adaptability towards redundant pathways [30,31,32]. This is further complicated where data within the same cell type have reported variation in ADAR1 dependency attributed to genetic drift [21,33]. It is important to address the pronounced variability in vulnerabilities to ADAR1 when interpreting data validating ADAR1 modulators. The domains of ADAR1 contribute to regulating different functions that impact cancer cell survival. The two ADAR1 isoforms share a common domain architecture consisting of a ZBDβ, three dsRBDs and the CDD (Figure 3). ADAR1p150 contains 295 additional amino acids in the N-terminus that contains the ZBDα and a nuclear export signal. Each of these domains contributes to ADAR1 function with distinct and sometimes complementary roles for controlling innate immune activation and RNA homeostasis.

A-to-I editing of dsRNA occurs within the CDD and can destabilize RNA duplexes and prevent their recognition by cytosolic sensors [34]. Both ADAR1 isoforms contain the CDD, but subcellular localization strongly influences substrate engagement. ADAR1 dominantly targets inverted Alu dsRNAs, which include variant repeats within telomeres that commonly form a G-quadruplex structure. The ZBDs of ADAR1 recognize these structures, particularly the Zβ domain, allowing editing of these mismatched sequences and preventing accumulation of R-loops, a structure made up of RNA:DNA hybrids, whose formations result in telomere instability [35]. ADAR1p110 can suppress RNA:DNA hybrid formation, but not cells expressing editing-deficient p110 (E912A) mutants or editing-active p150 isoforms, indicating that the editing activity of ADAR1p110 is essential [16]. In contrast, ADAR1p150 is specialized for IFN-stimulated cytoplasmic immune regulation [29]. A-to-I editing activity is critical for suppressing the MDA5-MAVS pathway, where unedited dsRNA promotes receptor oligomerization, interferon regulatory factor (IRF) activation, and type I IFN production (Figure 3) [29]. Editing-deficient mutants (e.g., E912A) that retain RNA binding ability, but lack catalytic activity, prevent MDA5-mediated signaling cascade, demonstrating that the RNA editing mechanism is critical for maintaining tolerance to immunogenic dsRNAs recognized by MDA5 [29]. A-to-I editing also reduces activation of other dsRNA sensors including OAS enzymes by destabilizing dsRNA [36]. The relative contribution of this pathway varies across cellular contexts: in some ADAR1 KO models cell lethality is rescued by depletion of RNaseL, the endonuclease triggered by OAS enzymes that leads to mRNA degradation and subsequently cell death, indicating the OAS pathway does mediate cell death upon ADAR1 loss [21,33].

The dsRBDs of ADAR1 are involved in editing-independent mechanisms that impact innate immune regulation. PKR is activated upon binding to dsRNA, leading to dimerization, autophosphorylation, and phosphorylation of eIF2α, ultimately resulting in translational arrest and induction of the integrated stress response (Figure 3) [37]. Use of RNA binding-deficient ADAR1 mutants, e.g., 3xEAA with the lysines in the conserved KKxAK motif altered in all three dsRBDs, and the E912A editing-deficient mutant, shows that both editing and dsRNA binding activities are important for PKR suppression [37]. The dsRBDs compete with other RNA binding proteins and immune sensors, like PKR, for binding of RNA substrates [37]. Beyond substrate competition, mutations like K778E and R790R in the third dsRBD can decrease direct PKR interaction blocking PKR suppression [37]. This interaction is RNA-dependent, as such binding of RNA substrate is impacted by the ability of the first and second dsRBD to effectively associate with RNA [37].

The Zα domain, unique to ADAR1p150, introduces additional mechanisms influencing immune suppression by sequestering and facilitating proper editing of left-handed Z-form RNA and through direct interactions with ZBP1 that lead to cell death pathways, i.e., PANoptosis (Figure 3) [38]. ADAR1 knock-in mice with Zα mutations (N175D and Y179A), which disrupt binding to Z-RNA, remain viable but exhibit elevated expression of ISGs [39]. This phenotype is dependent on ZBP1, as disruption of ZBP1’s Zα domains suppresses the ISG signature, indicating that aberrant ZBP1 recognition of these RNA substrates drives this signaling [39]. More severe consequences are observed in sensitized genetic contexts like *Adar1^mZα/-^* mice that have hemizygous expression of ADAR1 in combination with the Zα domain mutations that result in increased expression of endogenous retroelements (ERE)-derived transcripts, loss of editing of a subset of transcripts, and early postnatal lethality [39]. Lethality in this context is mediated through MDA5 signaling, supporting that Zα-mediated binding to Z-RNA is required for proper silencing of a subset of immunogenic transcripts [40]. Disruption of this interaction leads to accumulation of immunogenic Z-RNA species and activation of ZBP1 [38]. The Zα domain of ADAR1 also directly interacts with the Zα2 domain of ZBP1, preventing ZBP1 interaction with RIPK3 [38]. It is the combination of these complementary mechanisms that control ZBP1-mediated signaling and because ZBP1 contributes to ISG expression, which is also a potential modulation of other signaling pathways regulated by ADAR1.

ADAR1 regulates innate immunity with a combination of editing-dependent and -independent mechanisms including RNA destabilization, substrate competition, sequestration, and direct protein interactions. These overlapping mechanisms and pathways are important for interpreting results during therapeutic development. Perturbations targeting individual domains are unlikely to recapitulate the phenotype of ADAR1 knockout. Small molecule inhibitors that target catalytic activity may preferentially activate MDA5 without significantly activating PKR or ZBP1 pathways. Conversely, disruption of RNA binding or localization may differentially impact PKR or ZBP1 activation. In addition, unique nuclear functions of p110 suggest that compounds targeting the shared domains between isoforms may be more convoluted by differentially localized activities. Therefore, careful alignment of mechanisms, domain interaction, and phenotypic readouts such as interferon production, PKR phosphorylation, ZBP1 activation, or telomere integrity are important for interpreting effects of ADAR1 inhibition. With these mechanistic considerations in mind, we next discuss current ADAR1 modulators and therapeutic strategies being deployed to exploit these pathways.

## 3. Therapeutic Strategies Targeting ADAR1: Mechanisms, Advantages, and Limitations

A diverse range of strategies are being explored to therapeutically target ADAR1, spanning direct catalytic inhibition, RNA substrate-targeting approaches, and indirect modulation of ADAR1 expression and function. These approaches differ substantially in their mechanism of action, degree of target specificity, and potential clinical feasibility (Table 1). Importantly, the field has evolved from early, largely phenotypic observations of cytotoxicity readouts toward a more rigorous framework requiring biochemical validation, editing readouts, and mechanistic study of functional consequences. In the following section, we discuss the current landscape of therapeutic strategies utilizing ADAR1 or ADAR1 pathway modulation, highlighting the underlying mechanisms, therapeutic advantages, limitations, and challenges associated with each approach.

**Scheme 1:**
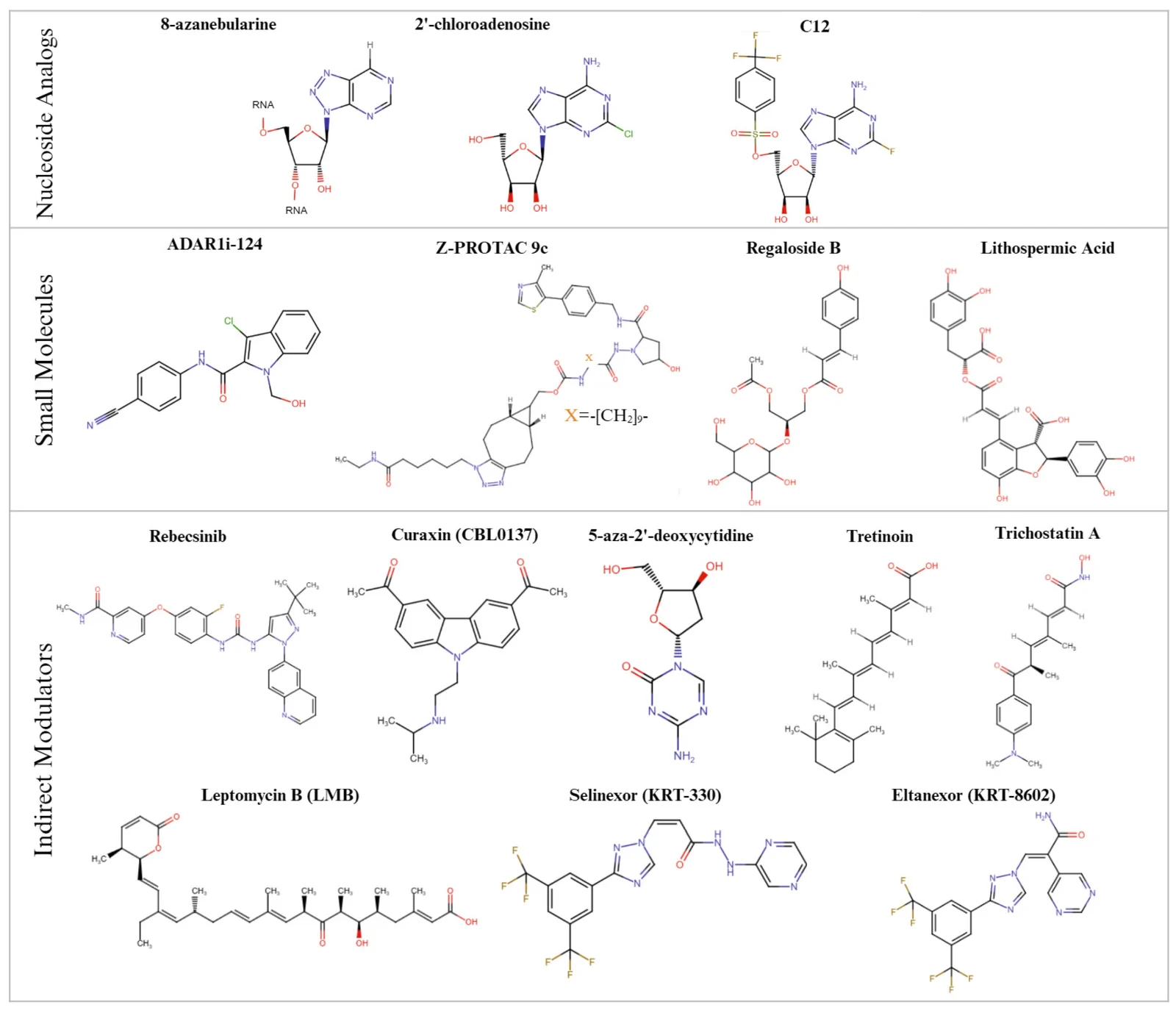
Compound structures of ADAR1 modulators. The structures shown correspond to the following representative compounds: 8-azanebularine [53], 2′-choloradenosine [42], C12 [42], ADAR1i-124 [43], Z-PROTAC 9c [47], regaloside B [54], lithospermic Acid [54], rebecsinib [22], curaxin (CBL0137) [49,50], 5-aza-2′-deoxycytidine [28,43], all-trans retinoic acid (tretinoin) [23,55], trichostatin A [56], leptomycin B (LMB) [57], selinexor (KRT-330) [45], and eltanexor (KRT-8602) [45]. Created in BioRender. Miller, W. (2026) https://BioRender.com/1shz6m5.

Early efforts to inhibit ADAR1 focused on nucleoside analogs that leverage the enzyme’s dependence on adenosine as a substrate. Chemical modification of the purine scaffold, particularly at the 8-position, produced compounds such as 8-azaadenosine, 8-chloroadenosine, and 8-azanebularine (Scheme 1). Initially these compounds were being used as probes of the ADAR2 active site in what became successful capture of X-ray crystal structures of the editing mechanism [51]. Mechanistically, their incorporation into dsRNA substrates can generate non-productive enzyme–substrate complexes, in which ADAR1 binds but is unable to complete hydrolytic deamination. For example, 8-azanebularine functions as a transition state mimic stabilizing the hydrated intermediate but lacking a proper leaving group; this effectively traps ADAR, preventing its editing activity [52]. Notably, this inhibitory effect is dependent on RNA duplex formation, as free nucleosides do not efficiently engage ADAR1, underscoring the importance of substrate context in enzyme inhibition. However, the subsequent evaluation of these compounds in cancer models highlighted a major challenge in ADAR1 drug discovery: cellular toxicity does not directly indicate selective target inhibition.

Despite the strong mechanistic principle, several widely cited nucleoside analogs have failed to demonstrate selective ADAR1 inhibition in cellular systems. Both 8-azaadenosine and 8-chloroadenosine exhibit antiproliferative effects across multiple cancer cell lines, yet they do not reduce A-to-I editing [41]. These compounds also do not mimic ADAR1 knockout activation of canonical dsRNA sensing pathway PKR; however, because this pathway is dominantly controlled by RNA editing-independent mechanisms, there is no sufficient evidence to support inactivity as a catalytic inhibitor could potentially leave those activities alone. However, their cytotoxicity persists in ADAR1-deficient systems; this indicates that their biological effects are ADAR-independent and likely to arise from broader disruptions in nucleotide metabolism or RNA processing [41]. Similarly, compounds derived from fludarabine such as ZYS-1 show potent cytotoxicity without clear biochemical evidence of ADAR1 inhibition [58,59]. Another compound, C12, derived from 2-chloroadenosine, has shown anti-tumor activities in xenograft models, reduced editing of GLI1 transcript, ADAR1 binding via surface plasmon resonance and good initial safety data (Scheme 1) [42]. Compared with earlier nucleoside analogs, these findings provide stronger evidence of potential ADAR1 specific activity by incorporating both target engagement and functional editing readouts. The primary advantage of this approach is that it leverages the endogenous substrate recognition requirements of ADAR1. However, several limitations and challenges still remain, including difficulty in achieving sufficient selectivity over other adenosine-dependent enzymes and metabolic pathways. Also, while reduced editing of individual transcripts supports true catalytic inhibition, a comprehensive analysis on global editing changes and a phenotypic pathway analysis would help support on-target ADAR1-mediated cell death. These findings underscore a critical conceptual shift in the field, i.e., anti-proliferative activity alone cannot be used as a proxy for target engagement, and careful integration of editing assays, binding studies, and pathway activation markers is essential for defining true ADAR1 inhibitors.

**Figure 4:**
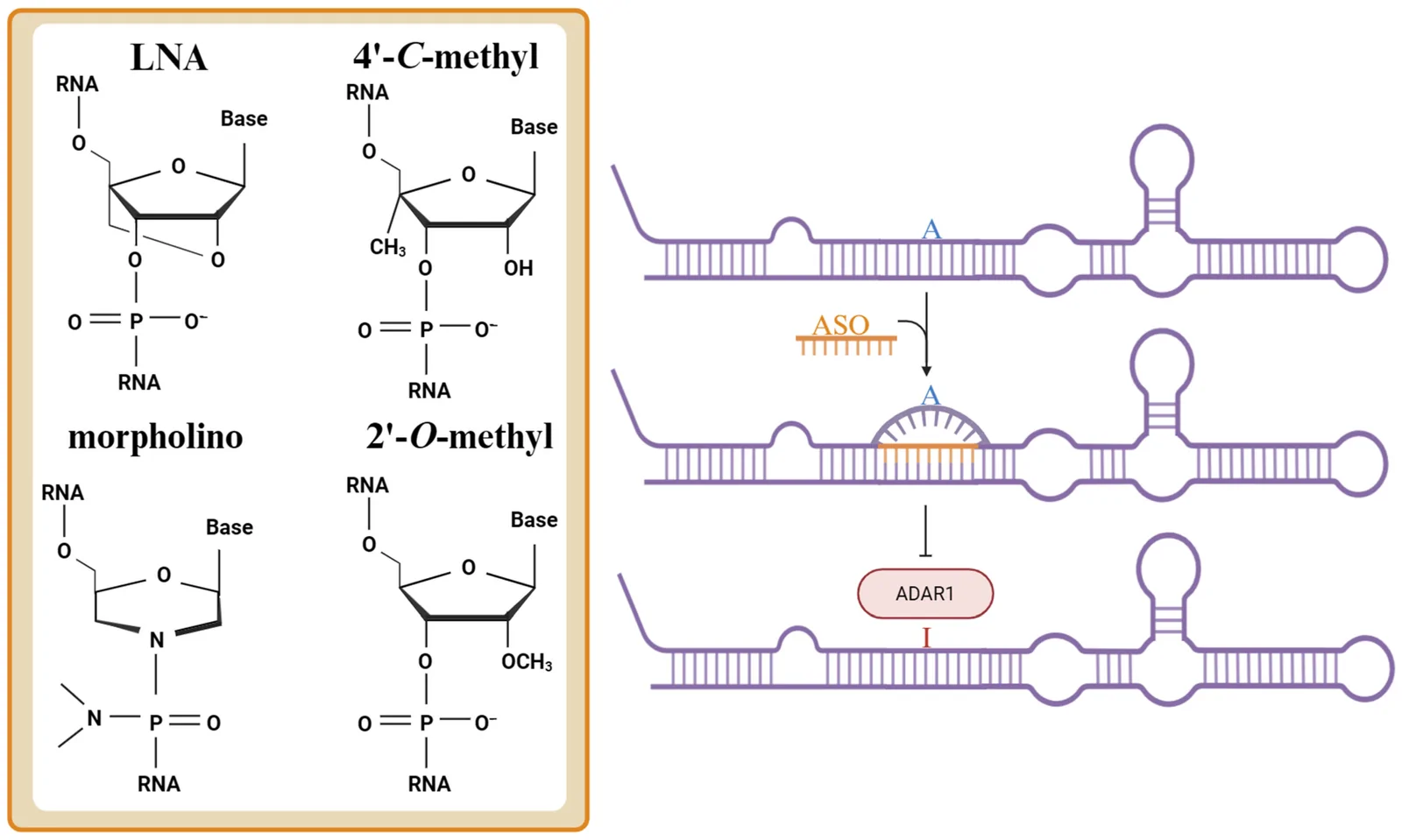
Modifications to the sugar and phosphate backbones of ADAR targeting ASOs and a visual representation of the strand invasion mechanism inhibiting ADAR1 editing. Created in BioRender. Miller, W. (2026) https://BioRender.com/1shz6m5.

Building on the limitations of early nucleoside analog approaches, other efforts are focused on rationally designing catalytic inhibitors with improved selectivity and validated on-target activity. Compounds such as ADAR1i-124 directly inhibit editing through the CDD, resulting in accumulation of unedited, immunostimulatory RNA species (Scheme 1 and Table 1) [43]. This accumulation activates innate immune sensors, MDA5 and ZBP1, resulting in increased IFN production and tumor cell death [43]. A major advantage of catalytic inhibition is the potential to selectively disrupt ADAR1’s RNA editing activity while preserving other functions of the protein, such as dsRNA binding. However, more study is needed as this may limit activation of pathways such as PKR signaling, providing a different immune signaling response compared to a full ADAR1 knockout. As far as establishing selectivity is concerned, direct catalytic inhibitors provide a clear framework for evaluating target engagement through measuring binding affinity, editing changes, and expression level changes in immune signaling proteins like MDA5. Several challenges face catalytic inhibitors. Cellular responses to catalytic inhibition remain context-dependent, influenced by factors such as ADAR1 isoform expression, baseline IFN signaling, the abundance of endogenous dsRNA, and the relative dependence of individual tumor types on ADAR1-mediated tumor suppression. These complexities highlight the need for better biomarker-driven approaches to apply these inhibitors to appropriate contexts and limit risks. In parallel, RNA-directed strategies are emerging as a highly specific alternative to protein-targeted strategies. Antisense oligonucleotides (ASOs) can be designed to selectively block editing at individual sites by targeting the editing complementary sequence and disrupting the dsRNA structure required for ADAR1 recognition. Through strand invasion, ASOs convert the target adenosine into a single-stranded conformation that is no longer suitable for deamination (Figure 4) [60]. Through this mechanism, ASOs function as substrate-level inhibitors, preventing ADAR engagement without needing to directly engage with the ADAR enzyme itself (Table 1).

The advantage of a substrate-level approach is the ability to selectively inhibit disease-associated editing events while preserving global ADAR1 activity. This may reduce the risk of systemic toxicity possible with broad ADAR1 inhibition and provide a high degree of sequence-level specificity. Notably, the effectiveness of this approach depends on precise control of RNA structure and sequence context, as well as chemical modifications that enhance stability, binding affinity, and cell uptake. Some of these modifications include locked nucleic acids (LNA), 4′-*C*-methyl, 2′-*O*-methyl, and morpholino backbone structures that improve targeting efficacy by increasing stability, selectivity, and cell uptake (Figure 4) [60]. This mechanism is also affected by ADAR1 sensitivity to local nucleotide context, particularly at the positions neighboring the edited adenosine, enabling rational design of ASO to exploit these preferences [52,61].

This strategy has demonstrated therapeutic potential in targeting oncogenic editing events such as those of AZIN1, where optimized ASOs achieve selective inhibition of AZIN1 editing resulting in suppressed tumor growth in vivo [44]. However, limitations remain, including challenges associated with efficient delivery to tumor tissues, the requirement for detailed characterization of pathogenic editing events, and limited applicability of identified tumor types with required oncogenic editing events that can alter tumor fate. In addition, compensatory changes in RNA processing or alternative editing events may limit the durability of this approach. Therefore, while ASOs offer exceptional specificity, their clinical application will likely depend on identification of tumors driven by well-defined ADAR1-dependent editing events.

Beyond direct inhibition, a growing number of indirect strategies aim to exploit the functional differences between ADAR1 isoforms including splicing modulators, nuclear export inhibitors (NEIs), Zα domain inhibitors, PROTACs, and other indirect ADAR1 pathway modulators (Table 1). These approaches utilize the modulation of ADAR1p150 expression, subcellular localization, or isoform-specific domains, with the goal of disrupting the IFN-induced immune regulatory functions of ADAR1p150 that occur in the cytoplasm (Table 1). The primary advantage shared with isoform-directed approaches is the potential to selectively disrupt the tumor-associated functions of ADAR1p150, which decreases the risks of adverse effects caused by inhibiting other ADAR enzymes. The isoform-directed approach may provide an improved therapeutic window compared to less selective ADAR targeting or total ADAR1 depletion. However, each strategy introduces unique advantages and limitations based on its specific mechanism of action.

One of such approaches involves specific isoform regulation at the level of pre-mRNA splicing. The small molecule rebecsinib (17S-FD-895) selectively suppresses production of the ADAR1p150 isoform through spliceosome targeting (Scheme 1 and Table 1) [22]. This results in reduced A-to-I hyper editing and impaired leukemic stem cell self-renewal, while sparing normal hematopoietic stem and progenitor cells in preclinical models [22]. As with all the ADAR1 isoform specific modulators, this strategy affects production of ADAR1p150. However, because rebecinib acts through splicing modulation rather than direct ADAR1 engagement, it remains challenging to evaluate the broader effects on RNA processing. The central role of the spliceosome regulates numerous cellular transcripts which raises potential concerns for off-target effects and long-term tolerability. Preclinical results show good initial safety and toxicity profiles, but further testing in different contexts and in clinical translation will be required [22]. Rebecsinib has received FDA approval to enter phase I clinical trials (NCT07250646).

A complementary strategy using nuclear export inhibitors (NEIs) targets ADAR1 subcellular localization, particularly the cytoplasmic enrichment of ADAR1p150 required for editing immunogenic dsRNA. Inhibition of nuclear export using XPO1 inhibitors like leptomycin B (LMB) and selinexor (KPT-330) reduce cytoplasmic ADAR1 levels, increase ZBP1 activation, and decrease tumor size (Scheme 1) [45]. The advantage to using NEIs is that it is exploiting the differential localization requirements of ADAR1 isoforms, preferentially disrupting the cytoplasmic regulatory functions of ADAR1p150, while potentially preserving ADAR1p110 activity. This strategy does not require direct engagement with ADAR1p150, so it may also overcome limitations of targeting the enzymatic site by also blocking editing-independent functions of ADAR1p150. While effective in principle, XPO1 regulates the export of numerous proteins and RNAs, raising safety concerns about broad disruption. Other NEIs like eltanexor (KPT-8602) (Scheme 1) show improved tolerability in patients and is currently under phase I clinical trial in combination with venetoclax for treatment of acute myeloid leukemia (NCT06399640).

To further decrease risk of off-target effects, efforts are also evaluating isoform-specific domain targeting, particularly focusing on the Zα domain unique to ADAR1p150. Small molecules such as AVA-ADR-001 disrupt Z-RNA binding, leading to the accumulation of immunogenic nucleic acids and increased IFN signaling including PKR, MDA5, and ZBP1 pathways that lead to translation inhibition and cell cycle arrest in cancer cells (Scheme 1 and Table 1) [46]. Mechanistically, AVA-ADR-001 contributes to ZBP1 activation by increasing its substrates and preventing its sequestration; in addition, ZBP1 upregulates ISGs like MDA5 and PKR which are also influenced by the increase in Z-conformation RNA reflecting the interconnected nature of RNA sensing mechanisms. The advantage of this strategy is that it is more specific than targeting the conserved CDD and should not interfere with the functions of the other conserved domains. The limitation of this strategy focuses more on the unknowns of cellular context: Is Zα domain needed? What is the expression of the downstream sensors? Are there compensatory pathways involved? Zα domain inhibitors have the prospect of being pan-cancer molecules, but their safety and efficacy in different contexts would need to be evaluated and would greatly benefit from assessment of different cell lines for ISG signatures and ZBP1 expression levels.

Targeted protein degradation is also being explored, leveraging ADAR1’s recognition of the Z-form nucleic acids. Z-DNA-based PROTACs (Z-PROTACs) link Z-DNA ligands to E3 ligase recruiters [47]. When ADAR1 binds to the Z-DNA substrate it comes into proximity to the E3 ligase recruited by the Z-PROTAC enabling selective degradation of ADAR1 [47]. Conceptually, this strategy is advanced because it utilizes substrate recognition rather than traditional binding pockets, providing an alternative approach for targeting a protein with complex RNA and nucleic acid interactions. Unlike the catalytic inhibitors that selectively block A-to-I editing, Z-PROTACs have the potential to remove editing-dependent and editing-independent functions of ADAR1p150, resulting in a cascade of apoptotic and necroptotic events mediated by MDA5 and ZBP1 [47]. The Z-PROTACs were able to specifically target ADAR1p110 and ADARp150 over other ADARs and ZBP1, supporting their potential for high specificity [47]. However, challenges remain for clinical translation, including optimization of cellular permeability, molecular stability, and in vivo delivery [47]. Additionally, because Z-PROTACs promote degradation of the entire ADAR1p150 protein, careful evaluation of toxicity from loss of essential ADAR1 activities in normal tissues will be required.

Other approaches seek to bypass ADAR1 entirely by modulating targets downstream of these immune pathways. Unlike direct ADAR1 inhibitors or modulators that impact ADAR1 expression or function, these strategies do not alter ADAR1 expression, localization, or enzymatic activity; they instead reproduce or regulate the downstream consequences of ADAR1 pathway disruption. For example, curaxin (CBL0137) activates immune signaling through ZBP1 in vivo, effectively recapitulating inhibition of ADAR1 Zα domain (Scheme 1) [49,50]. The advantage of this approach is that it can stimulate innate immune activation through ZBP1 without requiring ADAR1 dependency, but it does require reliance of the tumor on ZBP1. Compensatory pathways and variability in the activity of innate immune sensing pathways across tumor types may also influence the therapeutic response of this strategy.

In all, these studies illustrate a rapidly evolving landscape of ADAR1-targeting strategies, each with distinct advantages, limitations, and developmental challenges (Table 1). Crucial in these approaches is the tradeoff between specificity and systemic impact. Highly specific strategies such as ASOs and substrate-directed inhibitors offer precise control of individual editing events but require in-depth knowledge of disease-associated editing events and may have limited applicability across tumor types. In contrast, broader approaches like splicing modulation, nuclear export inhibition, and protein degradation strategies can more effectively disrupt ADAR1-dependent functions from achieving more global effects but at the potential cost of increased off-target risk. The most promising future directions lie in mechanistically precise, isoform specific interventions that target ADAR1’s role in tumor immune evasion while preserving its essential functions in normal tissues. Notably, the integration of these strategies into combination therapies has a lot of promise, as ADAR1 loss can resensitize numerous cell types to various chemo- and immuno-therapies [62,63].

## 4. Combination Therapies

ADAR1 modulators play a multifaceted role in activating innate immune sensing; combination with existing therapies is particularly promising to enhance anti-tumor efficacy. A unifying principle underlying these approaches is the amplification of immunogenic nucleic acid stress, achieved either by increasing burden of endogenous dsRNA or by sensitizing tumors to downstream IFN-mediated responses. As such, several studies combine agents for elevating dsRNA burden and increasing immune signaling with ADAR1 inhibition to increase therapeutic potency or sensitize resistant cancer cells [64,65].

Among the most well characterized combination treatments are DNA methyltransferase inhibitors (DNMTis), specifically 5-aza-2′-deoxycytidine (5-Aza-CdR) (Scheme 1). DNMTis induce widespread epigenetic reprogramming, leading to the reactivation of silenced ERVs and accumulation of ERV-derived dsRNAs [28]. This viral mimicry creates a cellular state that is highly dependent on ADAR1 editing to suppress aberrant immune activation. As a result, DNMTi treatment can convert otherwise ADAR1-independent tumors into ADAR1-dependent states, expanding the potential for ADAR1-modulated interventions [28,43].

This synergy has been demonstrated with catalytic inhibitors such as ADAR1i-124, where co-treatment with 5-Aza-CdR significantly enhanced anti-tumor activity. In addition, this combination reduced the IC_50_ of ADAR1i-124 by 12-fold and in an ADAR1-independent cell line resistant to ADAR1i-124 alone; the combination treatment induced a significant dose-dependent decrease in cell viability [43]. Mechanistically, the combination treatment increased immunogenic Z-RNA species, and upregulated MDA5 and ZBP1 cell death pathways [43]. These findings support a generalizable strategy for increasing endogenous dsRNA levels via DNMT inhibition to saturate ADAR1 editing capacity and upregulate ISGs, increasing sensitivity to ADAR1 inhibitors. Other epigenetic modulators and agents that induce transcription derepression of repetitive elements or genomic stability are also applicable.

Combinations with immune checkpoint blockades, particularly anti-PD-1 therapies, have shown significant promise. Genetic or pharmacological inhibition of ADAR1 can enhance tumor immunogenicity, where loss of ADAR1 in immunotherapy-resistant models could be resensitized to immunotherapy in vivo [25]. Consistent with this, AVA-ADR-001 demonstrated enhanced anti-tumor efficacy when combined with anti-PD-1 therapy, supporting that ADAR1 inhibition can prime tumors for immune-mediated clearance [46].

Additional combination strategies aim to directly enhance IFN signaling or disrupt tumor-intrinsic immune evasion mechanisms. Several studies have shown that exogenous IFN treatment or agents that increase endogenous IFN production can further elevate ISGs and dsRNA burden, increasing tumor dependency on ADAR1 [25,46]. Similarly, NEIs or induction of DNA damage and chromosomal instability can promote accumulation of aberrant nucleic acid species and promote ISG expression to boost IFN signaling pathways complementing ADAR-targeting strategies [16,45].

Together, these studies highlight that the most effective use of ADAR1-targeted therapies will be in combination regimens considering tumor-specific vulnerabilities in nucleic acid metabolism and immune signaling. Key determinants of response include the baseline level of ISG expression, the repertoire of dsRNA sensors expressed (e.g., MDA5, PKR, ZBP1), and the extent of ERV accumulation. As such, measurement of ADAR1-dependent biomarkers and immunogenic RNAs will help contextualize patients with the greatest applicability. In addition, these combination therapies allow an expansion of ADAR1 inhibitor application into cancers that are ADAR1-independent.

## 5. Clinical Translation of ADAR1 Therapeutic Strategies

Preclinical studies have established ADAR1 as a promising therapeutic target. Current clinical investigations are exploring multiple approaches, including direct pharmacological inhibition of ADAR1, modulation of ADAR1-associated immune responses, and evaluation of ADAR1 as a predictive biomarker for treatment response. These studies represent important steps toward defining the therapeutic potential of ADAR1-targeting and establishing biomarker-guided strategies for patient selection.

### 5.1. Nivolumab and All-Trans Retinoic Acid

The first clinical investigation exploring an ADAR1-associated vulnerability is an early phase study evaluating the combination of all-trans retinoic acid (ATRA) and nivolumab in patients with chemotherapy-refractory advanced or metastatic pancreatic adenocarcinoma (ID: NCT05482451) (Table 2). Preclinical studies support that ATRA can suppress ADAR1 expression and enhance ADAR1 immune-related signaling pathways, converting the pancreatic tumors into more immunologically responsive states [23,55]. Combination treatment with ATRA and anti-PD1 therapy led to improved anti-tumor activity compared with either treatment alone in pancreatic or breast tumor cells [23]. This study aims to determine whether ADAR1 expression can serve as a predictive biomarker for immunotherapy response and will also evaluate the therapeutic potential of combining ADAR1 modulation with immune checkpoint inhibition.

### 5.2. ADAR1 Expression Levels in Rectal Cancer

A second clinical investigation is evaluating ADAR1 as a biomarker of treatment response in patients with locally advanced rectal cancer undergoing total neoadjuvant therapy (TNT) (ID:NCT07108348) (Table 2). This is an observational registry study that aims to determine whether baseline ADAR1 expression and treatment-induced changes in ADAR1 expression correlate with response to chemotherapy and chemoradiotherapy. Prior studies report increased ADAR1 expression correlated with a reduction in overall survival, relapse-free survival, and uncreased recurrence after hepatic metastasectomy in metastatic colon cancer [66,67]. This study is evaluating tumor biopsies prior to and post-TNT for ADAR1 expression and looking for potential correlations with objective response rates, reoccurrence-free survival, and overall survival. This study addresses a very important translational question by evaluating whether ADAR1 expression can serve as a prognostic or predictive marker in a clinical setting.

### 5.3. Rebecsinib Phase 1 Clinical Trial

Rebecsinib (17S-FD-895), as a spliceosome modulator that inhibits ADAR1, is approved for a phase 1 clinical trial in patients with relapsed/refractory secondary acute myeloid leukemia or higher-risk myelofibrosis (ID: NCT07250646) (Table 2). This phase 1 study is designed for evaluation of safety, tolerability, and determination of maximum tolerated dose, along with biological activity readouts for response rates and outcomes of progression-free survival and overall survival. By modulating ADAR1 splicing and reducing specifically the ADAR1p150 isoform expression, rebecsinib represents an isoform-focused therapeutic strategy aimed at disrupting the suppression of immunogenic RNA sensing pathways by ADAR1p150 [22]. The assessments of rebecsinib in this trial for treatment response and safety will provide critical insights into the feasibility of targeting ADAR1 in humans.

### 5.4. Other Clinical Considerations

The identification of predictive biomarkers will be essential for successful clinical translation of ADAR1-related therapies because ADAR1 dependency varies substantially across tumor types and cellular contexts. ADAR1p150 is being considered as a biomarker; its elevated expression has been associated with altered immune profiles, including changes in immune checkpoint gene expression and immune cell infiltration, suggesting that ADAR1p150 expression may be a marker of tumor-immune state [65,68,69]. A standout for promising biomarker strategies is the assessment of interferon-stimulated gene (ISG) signatures [20,21,70,71,72]. ADAR1p150 is induced by interferon signaling, so tumors with elevated baseline ISG expression may represent a primed interferon state where those cancer cells are more dependent on ADAR1 for suppressing endogenous dsRNA sensing [20,21]. ISG expression scores have been reported as markers of inflammatory and elevated interferon states across multiple disease contexts including cancers and may provide a framework for identifying tumors with increased ADAR1 dependency [70,71]. ISG signatures are a good starting place for clinical translation; however, they are not sufficient to identify all ADAR1-dependent tumors, as successful activation of viral mimicry following ADAR1 inhibition also requires functional downstream signaling pathways, like MDA5/MAVS [29]. Due to this, future biomarker approaches will likely require an integrated assessment of multiple features including ADAR1 isoform expression, ISG activation, and genetic integrity of innate immune sensing pathways. The development and prospective validation of these biomarker strategies will be critical for selecting patients most likely to benefit from ADAR1-related therapies.

## 6. Conclusions and Future Perspectives

ADAR1 is a central regulator at the intersection of RNA biology, innate immune sensing, and tumor immune evasion. Through its capacity to catalyze A-to-I editing and editing-independent functions, ADAR1 maintains transcriptome integrity by preventing aberrant activation of cytosolic RNA sensors. In cancer, this homeostatic function is co-opted to suppress immunogenic dsRNA species derived from repetitive elements, e.g., ERVs, enabling tumor cell evasion of IFN-mediated surveillance. As a result, many tumors, especially those with high ISG signatures and high dsRNA burden, develop a functional dependency on ADAR1 for survival.

This dependency has positioned ADAR1 as a spotlight therapeutic target; however, effective targeting remains challenging. The presence of two key isoforms of ADAR1 introduces an additional layer of complexity. These isoforms differ in subcellular localization, domain composition, and biological function, with ADAR1p150 playing a dominant role in suppressing cytosolic dsRNA sensing, and ADAR1p110 primarily contributing to nuclear RNA processing and telomere-associated functions. These distinctions suggest that isoform selective modulation may offer a path toward improved therapeutic precision enabling disruption of tumor-specific immune evasion mechanisms while minimizing systemic toxicity risked by global RNA editing inhibition.

Efforts to pharmacologically target ADAR1 have yielded a diverse set of strategies, including catalytic inhibitors, RNA-binding disruptors, and substrate-directed approaches. While early small molecule inhibitors demonstrate proof-of-concept for targeting ADAR1 activity, challenges in selectivity remain a major barrier. Site-specific approaches like ASOs offer a complementary strategy by inhibiting editing only at specific editing sites. These approaches leverage structural and sequential preferences of ADAR1 to achieve targeted inhibition, although challenges related to delivery, stability and off-target effects are under further evaluation.

The clinical translation of targeted ADAR1 therapies will require careful consideration of patient selection and therapeutic context. As discussed throughout this review, ADAR1 dependency is highly context-dependent and influenced by tumor type, baseline interferon signaling, ADAR1 isoform expression, and the abundance of immunogenic dsRNA species. Emerging clinical studies are beginning to evaluate ADAR1 not only as a therapeutic target but also as a potential biomarker for predicting treatment response and identifying tumors most likely to benefit from ADAR1-related strategies. Importantly, the clinical potential of ADAR1 inhibition is most evident in the context of rational combination strategies. As discussed, ADAR1 limits the accumulation and immunogenicity of endogenous dsRNA. Therapies that increase dsRNA burden or IFN signaling, like epigenetic modulators, can therefore enhance tumor dependency on ADAR1. Combination of ADAR1 inhibitors with immune checkpoint blockade, i.e., anti-PD-1 therapies, has demonstrated the ability to amplify anti-tumor immunity by unmasking interferon responses mediated through immunogenic dsRNAs. These findings support a model in which ADAR1 inhibition serves not only as a standalone cytotoxic strategy, but as a sensitizing agent that potentiates immune response.

Looking forward, the successful clinical translation of ADAR1-targeting therapies will depend on several key advances. These include development of selective and potent inhibitors of ADAR1 or ADAR1 targets and the implementation of biomarker-driven patient stratification strategies that incorporate ADAR1 expression, ISG signatures and expression of immunogenic dsRNAs. Furthermore, development of these strategies can be rapidly accelerated by integrating computational approaches, including structure-based drug design and machine learning-guided predictions of RNA editing landscapes.

## Figures and Tables

**Table 1:** Overview of current ADAR1 pathway-related therapeutic strategies including summaries of mechanisms of action, advantages, limitations, developmental status, and representative studies.

Therapeutic Strategy	Representative Compounds /Approaches	Target/Mechanism of Action	Advantages	Limitations/Challenges	Developmental Stage	References
Nucleoside Analogs	C12 Series	Directly block catalytic turnover by trapping the active site.	May provide chemical scaffolds for further inhibitor development.	Early compounds lacked ADAR1 specificity and produced ADAR1-independent cytotoxicity; potential off-target effects on nucleotide metabolism.	Preclinical	[41,42]
Catalytic Inhibition	ADAR1i-124	Inhibit the catalytic deaminase domain of ADAR1, preventing A-to-I editing and increasing accumulation of immunostimulatory RNAs that activate innate immune signaling pathways.	Directly targets ADAR1 enzymatic activity; preserves non-catalytic functions that may reduce unintended activation of certain RNA sensing pathways.	Limited availability of potent and selective inhibitors; context-dependent whether tumor viability is strongly affected by editing loss.	Preclinical	[43]
Antisense oligonucleotides (ASOs)	ASOs targeting oncogenic ADAR1 targets, e.g., AZIN1.	Substrate level inhibitors that target the editing complementary sequence of specific target sites to disrupt the dsRNA structure necessary for ADAR1 recognition.	High target specificity; prevents ADAR1 engagement without requiring a disturbance of ADAR1 itself; does not disturb global editing levels.	Delivery is challenging and requires high stability. Highly specific design is necessary for effective stability and selectivity and is limited to known pathogenic editing events.	Preclinical	[44]
Splicing Modulators	Rebecsinib (17S-FD-895)	Inhibits ADAR1 splicing into ADAR1p150.	Provides potential isoform selectivity by preferentially reducing ADAR1p150 protein expression while preserving functions of ADAR1p110. Pre-IND studies report favorable safety profile.	Hard to distinguish whether therapeutic effects arise specifically from ADAR1 pathways or from more broader alterations in RNA processing/splicing.	Clinical	[22]
Nuclear Export Inhibitors (NEIs)	Eltanexor (KPT-8602)	Prevent nuclear export by exportin-1 (XPO1) of ADAR1p150, reducing ADAR1p150 expression levels in the cytoplasm and decreasing editing of immunogenic dsRNAs.	Exploits the differential localization of the two ADAR1 isoforms.	XPO1 controls export of other proteins and RNAs that could have potential toxicity. Lack of ADAR1 specificity due to broad XPO1 cargo regulation.	Preclinical/Clinical	[45]
Zα Domain Inhibitors	AVA-ADR-001	Target the Z alpha domain to disrupt Z-DNA/Z-RNA recognition by ADAR1p150 and sequestration of ZBP1. Promotes accumulation of ZBP1 activating RNAs.	Selectively targets ADAR1p150-associated immune regulatory functions.	The contributions of editing-independent Zα functions to tumor survival remain incompletely defined.	Preclinical and early translational development.	[46]
PROTACs	Z-PROTAC 9c	PROTAC is attached to ADAR1-specific Z-DNA. ADAR1 recognition of the Z-DNA results in E3 ligase recruitment and reduced ADAR1 protein levels through proteasomal degradation.	Complete target removal overcomes limitations of catalytic-only inhibition by also impacting editing-independent functions.	Challenges with cell permeability; systemic depletion may affect normal tissues.	Early preclinical	[47]
Indirect Pathway Modulation	Curaxin (CBL0137)	Modulate targets in RNA sensing pathways downstream of ADAR1 to increase immune activation.	Mimic ADAR1 loss; strong combination therapy candidates for existing anti-PD1 therapies and epigenetic modulators.	Reduce specificity for ADAR1-dependent tumors; may not fully reproduce ADAR1 inhibition; unknown pathway redundancy.	Preclinical/Clinical	[48,49,50]

**Table 2:** Current ADAR1 clinical explorations.

Study	Therapeutic Strategy	Cancer Type	ADAR1 Relevance	Study Design	References
NCT05482451	ATRA + nivolumab	Chemotherapy-refractory advanced or metastatic pancreatic adenocarcinoma	ATRA-mediated suppression of ADAR1 is proposed to enhance immune checkpoint blockade response.	Interventional-Early Phase 1	[23,55]
NCT07108348	ADAR1 expression monitoring during TNT	Locally advanced rectal cancer patients	Evaluates ADAR1 expression as a biomarker of treatment response and prognosis for TNT response.	Observational (Patient Registry)	[66,67]
NCT07250646	Rebecsinib (17S-FD-895)	Relapsed or refractory secondary acute myeloid leukemia or higher-risk myelofibrosis	Spliceosome modulation inhibiting expression of ADAR1p150 isoform.	Interventional-Phase 1	[22]

## Data Availability

No new data were created or analyzed in this study. Data sharing is not applicable to this article.

## Abbreviations

ADAR, adenosine deaminase acting on RNA; PKR, protein kinase R; MAVS, mitochondrial antiviral signaling; MDA5, melanoma differentiation-associated protein 5; OAS, oligodenylate-synthetase; ISG, interferon stimulated gene; PROTACs, proteolysis targeting chimeras; dsRNA, double-stranded RNA; CDD, catalytic deaminase domain; NSCLC, non-small-cell lung cancer; ISG, interferon stimulated gene; IFN, interferon; ZBD, Z-DNA binding domain; ZBP1, Z-DNA binding protein 1; ASOs, antisense oligonucleotides; LMB, leptomycin B; Z-PROTACS, Z-DNA-based PROTACS; 5-Aza-CdR, 5-aza-2′-deoxycytidine; DNMTis, DNA methyltransferase inhibitors; NEIs, nuclear export inhibitors; ERVs, endogenous retroviruses.
